# Parkinson’s Syndrome After Cranial Radiotherapy: A Case Report

**DOI:** 10.7759/cureus.24411

**Published:** 2022-04-23

**Authors:** Kati K Reddy, Mark D Anderson, Srinivasan Vijayakumar, Toms Vengaloor Thomas

**Affiliations:** 1 Radiation Oncology, University of Mississippi Medical Center, Jackson, USA; 2 Neuro-Oncology, University of Mississippi Medical Center, Jackson, USA

**Keywords:** low grade gliomas, atypical parkinsonian disorders, chemo radiotherapy (chemo-rt), neuro oncology, radiation & medical oncology

## Abstract

Parkinson’s syndrome is a group of signs and symptoms where the core issue is bradykinesia and could be a manifestation of idiopathic parkinsonism (Parkinson's disease, PD), secondary parkinsonism, or parkinsonism due to neurodegenerative disease. PD is the most common cause of Parkinson's syndrome, accounting for approximately 80% of cases. The secondary causes of Parkinson's syndrome include tumors, trauma, hydrocephalus, chemotherapy, medications including amphotericin B, metoclopramide, and radiation treatment. Parkinsonian symptoms secondary to radiation treatment are rarely reported in the literature and are usually not alleviated by carbidopa-levodopa. This report describes a 64-year-old man diagnosed with low-grade astrocytoma of the midbrain who developed unilateral parkinsonian symptoms one year after chemoradiation treatment. This case report also sheds further light on the details of reported cases and the treatment options through an extensive literature review. Clinicians need to be aware of patients developing this rare complication following radiation treatment.

## Introduction

Parkinson’s syndrome is a group of signs and symptoms where the core issue is bradykinesia and could be a manifestation of idiopathic parkinsonism (Parkinson's disease, PD), secondary parkinsonism, or parkinsonism due to neurodegenerative disease. PD is the most common cause of Parkinson's syndrome, accounting for approximately 80% of cases. It occurs due to the progressive degeneration of dopaminergic neurons in the substantia nigra. The majority of cases (85-90%) have no clear causes; however, 10-15% of patients have a positive family history. The etiology of Parkinson's disease is still under investigation. The secondary causes of Parkinson's syndrome include tumors, trauma, hydrocephalus, chemotherapy, medications including amphotericin B, metoclopramide, and radiation treatment [1-15]. Parkinsonian symptoms secondary to radiation treatment are rarely reported in the literature and are usually not alleviated by carbidopa-levodopa.

This report describes a 64-year-old gentleman who developed unilateral Parkinsonian symptoms one year after chemoradiation treatment for low-grade astrocytoma of the midbrain.

## Case presentation

**Figure 1:**
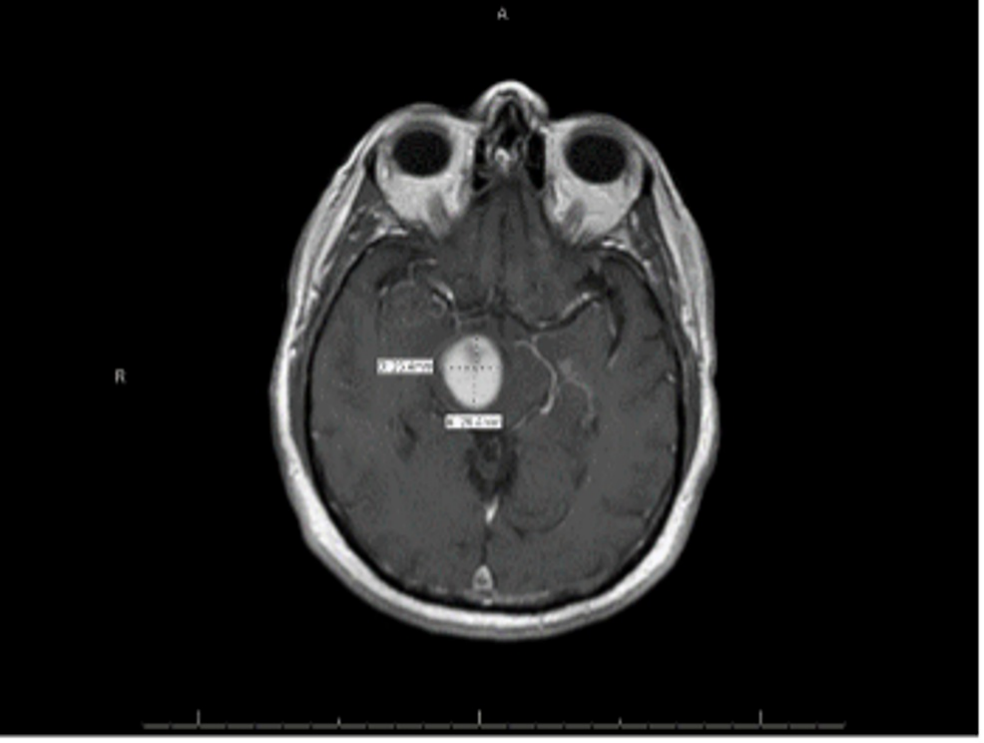
MRI findings at the time of presentation – homogenously enhancing solitary mass centered in the right midbrain and right cerebral peduncle measuring 2.3 cm × 2.6 cm × 2.5 cm

**Figure 2:**
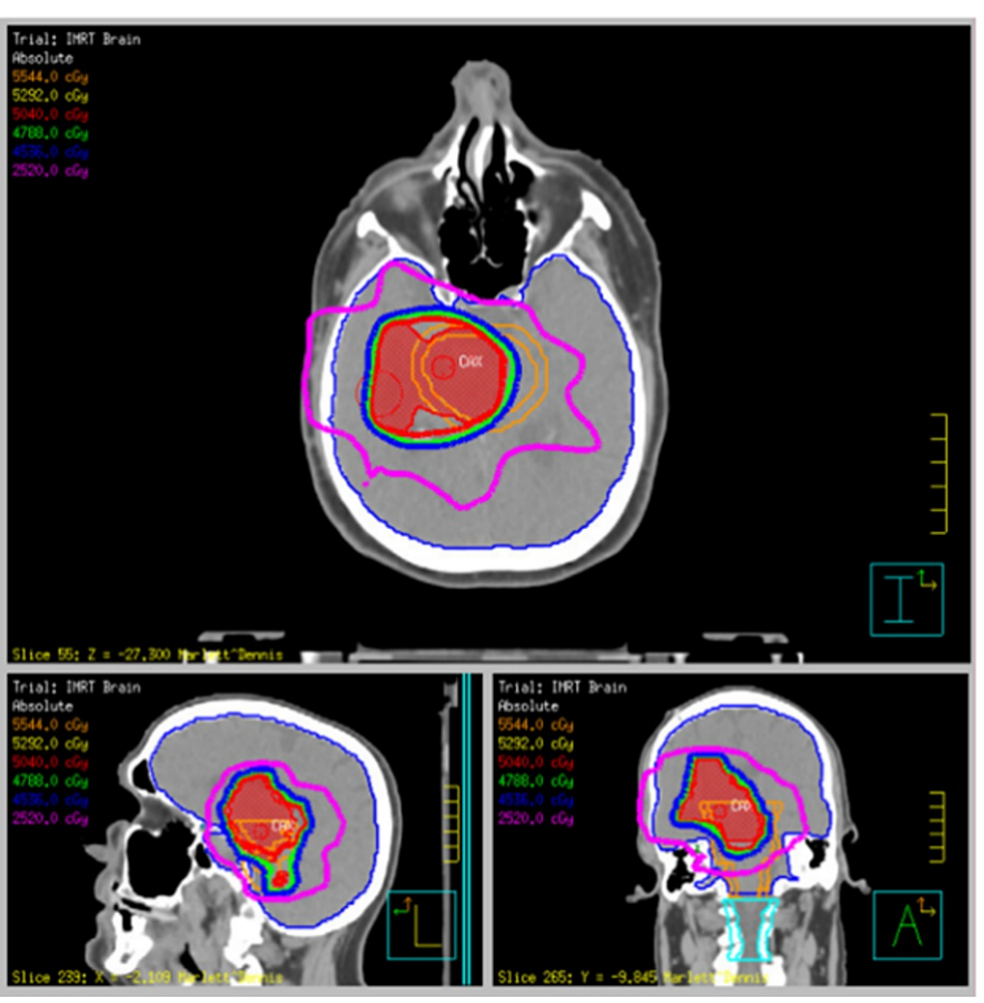
Radiation treatment plan - 5040 cGy at 180 cGy daily for 28 fractions using intensity-modulated radiation treatment

Our patient, a 64-year-old Caucasian male, presented to the hospital with progressive left-side weakness. He underwent magnetic resonance imaging (MRI) of the brain, which revealed a homogeneously enhancing mass centered in the right midbrain and right cerebral peduncle measuring 2.3 cm × 2.6 cm × 2.5 cm. The non-enhancing adjacent T2/FLAIR signal abnormality extended into the posterior limb of the internal capsule cranially and posterior pons caudally (Figure 1). He underwent a stereotactic biopsy of the lesion, and the pathology revealed a low-grade astrocytoma. The patient underwent definitive chemoradiation treatment with 5040 cGy at 180 cGy daily for 28 fractions using intensity-modulated radiation treatment (IMRT) concurrently with Temozolomide 75 mg/m^2^ (Figure 2).

The follow-up MRI after the treatment was indicative of the progression of the disease. He was started on Bevacizumab and Temozolomide, and his disease remained stable on the successive scans. He was tolerating the treatment well. One year later, during his routine follow-up visit, the patient reported a new involuntary movement in the left arm for two weeks. He noted that the movements started in the thumb and progressed over two weeks, affecting the whole left hand and part of the arm. The physical examination revealed 5/5 strength in the left proximal upper arm, 3/5 in the left distal arm, and 4/5 in the left leg with increased tone and decreased bulk. Continuous rhythmic movements in the left thumb and hand were noticed, which were only mildly suppressible. It did not involve the face or the leg. He underwent further workup with an MRI of the brain, which revealed that the glioma was stable. The symptoms were concerning for Parakinsonian tremors.

**Figure 3:**
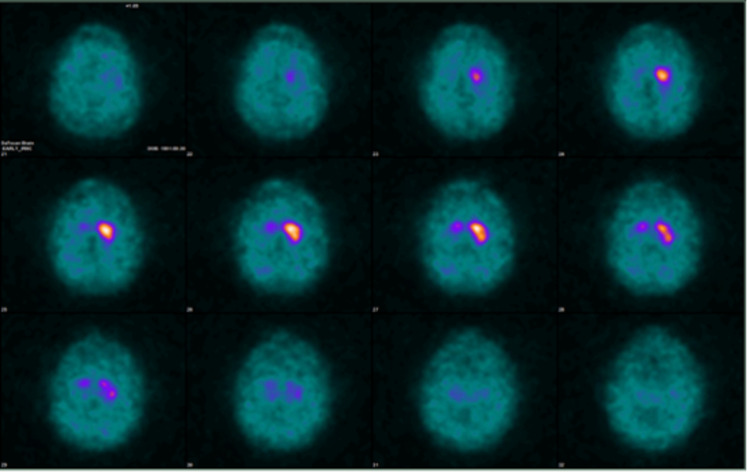
DATscan with decreased uptake in right caudate and absent uptake in right putamen, consistent with Parkinson’s disease. There was normal comma-shaped activity in the left caudate and left putamen.

Further evaluation with DATscan revealed decreased uptake in the right side caudate and absent uptake in the right putamen, consistent with Parkinson’s disease. There was regular comma-shaped activity in the left caudate and left putamen (Figure 3). For the treatment of tremors, the patient was started on carbidopa-levodopa with complete resolution of the involuntary movement. 

## Discussion

This report describes a 64-year-old gentleman who developed unilateral parkinsonian symptoms one year after chemoradiation treatment for low-grade astrocytoma of the midbrain. The MRI of the brain did not reveal any progression of the glioma. The nuclear medicine scan, DATscan, reported the hypoactivity of the right caudate nucleus and the absence of activity in the right putamen.

Radiation treatment is known to cause several acute and chronic changes in the brain through various mechanisms, and it is an active area of investigation [16-22]. Given the location of the midbrain tumor and the area that received radiation therapy, the pathophysiology likely closely mimics the loss of the substantia nigra in idiopathic Parkinson’s disease.

A literature review on secondary parkinsonism caused by radiation treatment to the brain revealed six cases reported in the literature, including two pediatric and four adult patients [8-14]. These patients did not significantly respond to levodopa therapy, presumably due to alternate mechanisms of injury.

One case reported the circumstances of a 14-year-old female with craniopharyngioma who underwent surgery followed by adjuvant radiation treatment with 5400 cGy using 180 cGy daily fractions with an IMRT plan [11]. The patient started having parkinsonian symptoms three weeks after finishing radiation treatment. The patient was started on levodopa but did not have any symptomatic improvement. At that point, levodopa was stopped, and a dopamine agonist was initiated, and she responded to that treatment. The patient’s symptoms improved 12 months after finishing radiation but had persisting symptoms at the time of the report.

Another case is of a 16-month-old female patient with a recurrence of the posterior fossa tumor who underwent surgery of the posterior fossa lesion followed by craniospinal radiation treatment to 3500 cGy in 21 fractions followed by a 2000 cGy boost to the posterior fossa in 12 fractions [11]. The patient developed Parkinsonian symptoms seven months after finishing radiation treatment. The imaging confirmed bilateral signal changes in the basal ganglia. She was started on benztropine, and the symptoms have improved. The patient is doing well, without any recurrence of disease or Parkinsonian symptoms for five years, at the time of the case report.

There are a few adult patients with reported cases of secondary Parkinson’s syndrome caused by radiation treatment. A 72-year-old man diagnosed with glioblastoma underwent surgical resection and adjuvant radiation treatment, followed by two infusions of cisplatin and then 12 cycles of Temozolomide every three weeks for 12 weeks [9]. During the radiation treatment, he developed a progressively worsening shuffling gait, motor slowness, and left-side weakness. Once he finished all his chemotherapy, he was evaluated at the movement disorder clinic. The clinical examination revealed generalized spasticity with superimposed rigidity, decreased left-hand movement with bradykinesia, and hyperreflexia. The MRI brain showed a marked increase in diffuse white matter intensity, worse on the right, consistent with post-radiation encephalopathy. A levodopa trial was performed without any improvement in the patient's symptoms. The history and exam were consistent with Parkinsonism secondary to radiation treatment. The patient progressively deteriorated and deceased within one year of evaluation at the movement disorder clinic. One patient developed secondary Parkinson's syndrome during radiation treatment, while another patient developed symptoms seven years after treatment, and the fourth patient developed symptoms 39 years after radiation treatment [8,12-14]. All these patients were resistant to carbidopa-levodopa treatment for the resolution of their symptoms. In our patient’s case, carbidopa-levodopa treatment successfully resolved his tremors.

## Conclusions

Secondary Parkinson's syndrome is a rare complication of radiation treatment of the basal ganglia. Clinicians need to be aware of patients developing this rare complication following radiation treatment. It may be beneficial for physicians to avoid radiation to the basal ganglia if and whenever possible. Furthermore, it is best to counsel the patient about this rare complication if the target is near the basal ganglia. Further studies are needed to investigate the basal ganglia's radiation pathophysiology.
